# CAR-T cell therapy in glioblastoma: from αβ to γδ T-cell platforms

**DOI:** 10.3389/or.2026.1808229

**Published:** 2026-07-02

**Authors:** Costanza Dieli, Roberta Costanzo, Anna Maria Corsale, Federica Paolini, Marta Di Simone, Claudia Avellone, Francesco Dieli, Domenico Gerardo Iacopino, Salvatore Marchiafava, Rosario Maugeri, Serena Meraviglia

**Affiliations:** 1 Central Laboratory of Advanced Diagnosis and Biomedical Research (CLADIBIOR), University of Palermo, Palermo, Italy; 2 Department of Precision Medicine in Medical, Surgical and Critical Care (MePreCC), University of Palermo, Palermo, Italy; 3 Developmental Biology and Cancer (DBC), UCL Great Ormond Street Institute of Child Health, London, United Kingdom; 4 Neurosurgery Unit, “Villa Sofia” Hospital, Palermo, Italy; 5 Department of Biomedicine, Neuroscience and Advanced Diagnosis (BiND), University of Palermo, Palermo, Italy; 6 Neurosurgery Unit, AOUP “Paolo Giaccone”, University of Palermo, Palermo, Italy; 7 Department of Health Promotion, Mother and Child Care, Internal Medicine and Medical Specialties (ProMISE), University of Palermo, Palermo, Italy

**Keywords:** alpha/beta T-cell, CAR-T cells, gamma delta T-cell, glioblastoma, immunotherapy

## Abstract

Glioblastoma multiforme (GBM) remains a highly aggressive brain tumor with limited treatment options and poor survival. The persistent clinical failure has driven interest in novel, immune-based strategies with higher specificity and greater cytotoxic potency, among which chimeric antigen receptor (CAR)-T-cell therapy has emerged as a leading approach. αβ CAR-T cells demonstrate safety but limited efficacy due to antigen heterogeneity, an immunosuppressive tumor microenvironment (TME), and restricted trafficking to the central nervous system (CNS). γδ CAR-T cells, combining innate major histocompatibility complex (MHC)-independent tumor recognition with engineered antigen specificity, have demonstrated preclinical cytotoxicity and features consistent with intratumoral persistence in selected models, as well as off-the-shelf potential, making them a next-generation immunotherapy strategy for GBM.

## Introduction

GBM is the most common and aggressive primary malignant brain tumor in adults, accounting for more than 50% of all malignant GBMs and with an annual incidence of approximately 3-5 per 100,000 patients (1). Despite multimodal therapy, maximal safe surgical resection followed by radiotherapy with concomitant and adjuvant temozolomide, prognosis remains poor, with a median overall survival of 14–18 months and a 5-year survival of less than 10% (1–3). Several factors contribute to the poor outcome, including its infiltrative growth pattern, profound inter- and intra-tumoral heterogeneity, and the presence of therapy-resistant GBM stem cells (GSCs) which exhibit enhanced DNA repair capacity, multidrug resistance and abilities to drive recurrence even after aggressive treatment (1, 3). Moreover, the TME plays a critical and, mostly, pivotal role in treatment resistance. In fact, it is characterized by extensive immunosuppression due to high levels of inhibitory cytokines, paucity of effector T-cell infiltration, and abundant immunosuppressive cells (e.g., regulatory T-cell, tumor-associated macrophages). These features not only foster tumor progression but also severely limit the effectiveness of immunotherapeutic strategies (1, 3). Given the limited efficacy of current therapies, there is an obvious increasing need for innovative and effective treatment modalities. Among emerging approaches, CAR-T-cell therapy has gained substantial interest. CAR-T cells are genetically engineered autologous T lymphocytes that express synthetic receptors capable of recognizing tumor-associated antigens in an MHC-independent manner. Their transformative success in hematologic malignancies, particularly CD19-directed CAR-T therapies achieving high response rates in relapsed/refractory B-ALL and lymphoma (3), enables translation to various solid tumors, including GBM (4).

Several clinical trials are currently underway to assess its safety and therapeutic potential in this solid tumor setting. This systematic review focuses specifically on CAR-T-cell therapy for GBM, analyzing data from ongoing and completed clinical studies and discussing the key benefits and challenges of its use. By integrating recent findings, this review aims to provide a comprehensive overview of CAR-T-cell therapy, both αβ and γδ T-cell platforms, in GBM, and its potential to reshape future treatment strategies for this aggressive cancer.

## Clinical therapeutic approaches in GBM

The standard of care for newly diagnosed GBM is built on a multimodal framework that combines maximal safe resection, radiotherapy, and temozolomide (TMZ), yet these interventions remain largely palliative and fail to meaningfully alter the natural history of the disease. Median overall survival rarely exceeds 14–16 months, and 5-year survival remains below 10% despite optimal guideline-concordant treatment (5–7). Surgical resection is the first therapeutic intervention, aiming to achieve cytoreduction, symptomatic relief and improved efficacy of subsequent chemotherapy and radiation. Large retrospective cohorts and prospective series consistently show a survival advantage with more extensive resection, including supratotal strategies when safely feasible. However, due to its infiltrative growth pattern, GBM cannot be fully eradicated surgically and residual tumor cells inevitably seed recurrence (6, 8, 9). Even in optimal scenarios, recurrence occurs within months, commonly at the margins of the resection cavity, highlighting the limitations of surgery as a curative modality. Following resection, patients undergo radiotherapy with concomitant TMZ, a paradigm established by the landmark Stupp trial, which demonstrated improved survival compared to radiotherapy alone. Median OS improved from 12.1 to 14.6 months, with a notable 5-year survival benefit in a small subset of patients. O6-methylguanine-DNA methyltransferase (MGMT) promoter methylation was identified as a critical biomarker of response to alkylating therapy, and its assessment has since become a standard component of therapeutic decision-making (6, 8, 9).

Nevertheless, patients with unmethylated MGMT derive minimal benefit from TMZ, yet few alternative first-line systemic options exist, leaving a substantial proportion of patients undertreated from a molecular perspective. In the adjuvant phase, tumor-treating fields (TTFields) have been incorporated into clinical practice following evidence of improved progression-free and overall survival when combined with maintenance TMZ. In a randomized study, TTFields extended median OS to approximately 20 months versus 15.6 months with TMZ alone, particularly benefiting younger and higher-performing patient subgroups (6, 8, 9). Although adoption remains variable, TTFields represent one of the few advancements in first-line therapy in nearly two decades. Despite these interventions, recurrence is nearly universal, and treatment of recurrent GBM remains an area of significant unmet clinical need. Recurrence typically occurs locally, although distant and multifocal failures are increasingly recognized due to refined imaging. For selected patients with good performance status and circumscribed recurrence, repeat surgical resection may provide symptomatic relief, facilitate cytoreduction, and enable tissue acquisition for molecular profiling. However, survival benefits are modest and patient selection is critical (6, 8, 9).

Re-irradiation strategies, including stereotactic radiosurgery and hypofractionated approaches, can provide local control in well-selected cases, though risks of radio necrosis and cumulative toxicity limit their widespread application. Response rates remain modest and durability limited, reflecting inherent radioresistance and the aggressive biology of recurrent GBM (5, 6). Systemic therapies in the recurrent setting are similarly disappointing. Bevacizumab, a VEGF-targeting monoclonal antibody, received accelerated approval based on radiographic response rates (∼35%) and improvement in peritumoral edema, yet randomized trials have not demonstrated an overall survival benefit, and responses are transient. Nitrosoureas such as lomustine remain widely used but typically yield progression-free survival of 3–4 months or less. TMZ rechallenge, platinum agents, and combination regimens have been explored, but their impact on survival is minimal and toxicity can be significant.

The past decade has brought enthusiasm for targeted therapies, driven by improved understanding of molecular drivers including EGFR amplification/variant III mutation, PTEN loss, PDGFRA alterations, and dysregulation of the PI3K-AKT-mTOR pathway. However, clinical translation has been largely disappointing. mTOR inhibitors, PI3K inhibitors, and multi-kinase inhibitors have shown limited clinical efficacy due to intratumoral heterogeneity, pathway redundancy, and insufficient blood–brain barrier penetration (5, 7). Similarly, therapies targeting GSCs, despite strong preclinical rationale, have not yielded durable clinical responses, partly due to phenotypic plasticity and evolution under therapeutic pressure (10, 11).

## Novel and immune-based therapeutic strategies

Parallel efforts in immunotherapy have also produced mixed results. Vaccine-based strategies (peptide, dendritic cell, neoantigen vaccines) demonstrate immune activation but only modest clinical benefit. Immune checkpoint inhibitors such as anti-PD-1 agents have not shown meaningful efficacy in unselected GBM patients, reflecting the tumor’s profoundly immunosuppressive microenvironment and low neoantigen burden. Oncolytic virotherapy has shown safety and occasional prolonged responses but lacks a consistent survival benefit (10, 11). Even in clinical trials, objective response rates remain low and improvements in survival are incremental at best. As a result, contemporary management of GBM remains fundamentally supportive and palliative, with current modalities offering only temporary disease control. Median overall survival after recurrence is approximately 8 months, and long-term survival is exceedingly rare (2). The confluence of infiltrative tumor biology, therapy-resistant GSC populations, molecular heterogeneity, immunosuppression, and the restrictive blood–brain barrier collectively undermines the efficacy of traditional and emerging therapies. This persistent clinical failure has driven interest in novel, immune-based strategies with higher specificity and greater cytotoxic potency, among which CAR-T-cell therapy has emerged as a leading approach.

Several characteristics of GBM provide a rationale for exploring CAR-T therapy in this setting. First, multiple surface antigens, such as IL13Rα2**,** EGFRvIII**,** HER2**,** GD2**,** EphA2**,** and B7-H3, are highly expressed in GBM and minimally represented in healthy brain tissue, enabling selective targeting and reducing the risk of on-target/off-tumor toxicity (1, 3). More recently, GD2 has emerged as an additional target of interest in GBM, with preclinical studies using advanced human-relevant models, such as brain stem organoids, highlighting both antitumor activity and key limitations related to CAR-T-cell persistence and functional exhaustion within the CNS microenvironment (12).

Second, preclinical studies have demonstrated compelling antitumor activity, including effective killing of GSCs, tumor regression in murine intracranial GBM models, and prolongation of survival in xenograft models (3). Notably, IL13Rα2- and EGFRvIII-directed CAR-T cells demonstrated potent cytotoxicity against heterogeneous GBM populations, suggesting the potential to overcome antigenic diversity, a major barrier to therapy (1, 3). Early clinical studies further demonstrated the feasibility and safety of CAR-T infusion in GBM patients. A meta-analysis of clinical trials in recurrent GBM reported a favorable toxicity profile, with low rates of cytokine release syndrome (CRS ≤ grade 2 in 9.5% of patients) and uncommon neurotoxic events (25% grade ≤2), confirming that CAR-T therapy is generally well tolerated in this population (2). However, clinical effectiveness has been limited. The pooled objective response rate (ORR) across available trials was only about 5%**,** with a median overall survival of approximately 8 months following treatment (2).

Barriers include the blood-brain barrier (BBB), antigen heterogeneity leading to immune escape, limited persistence of CAR-T cells within the TME, and profound local immunosuppression. Nevertheless, certain individual cases, such as complete regressions observed with IL13Rα2-directed CAR-T administered via intrathecal or intracavitary routes, support the potential of optimizing delivery strategies to enhance therapeutic efficacy (1, 3). Thus, ongoing research focuses on improving antigen specificity, developing multivalent or bispecific CAR constructs, optimizing locoregional delivery to bypass the BBB, and combining CAR-T therapy with agents that modulate the immune microenvironment.

In particular, the BBB represents one of the principal obstacles to the effective treatment of GBM, severely limiting the penetration and accumulation of therapeutic agents within the tumor microenvironment. Although the abnormal vasculature associated with GBM may partially increase permeability in selected tumor regions, the BBB and the related blood–brain tumor barrier (BBTB) continue to restrict the delivery of most chemotherapeutic agents and biologics. Consequently, significant research efforts have focused on the development of advanced drug delivery systems capable of enhancing transport across these physiological barriers while improving tumor specificity and reducing systemic toxicity. Recent studies have highlighted nanotechnology-based delivery platforms as particularly promising tools to address these limitations.

Among the most extensively investigated strategies, lipid-based nanoparticles have attracted considerable attention due to their biocompatibility, biodegradability, and capacity to encapsulate both hydrophilic and hydrophobic compounds. In parallel, inorganic nanoparticles have demonstrated substantial potential for BBB penetration and multimodal therapeutic applications. Gold nanoparticles, iron oxide nanoparticles, mesoporous silica nanoparticles, dendrimers, and quantum dots exhibit unique physicochemical properties that enable controlled drug release and radiosensitization (13, 14). Additionally, hybrid biomimetic systems combining inorganic cores with lipid membranes are increasingly being investigated to improve biocompatibility and BBB crossing efficiency (13, 14). Overall, nanotechnology-based therapeutic platforms represent a rapidly evolving field with substantial potential to overcome BBB-associated limitations in glioblastoma therapy. However, despite these advances, challenges related to long-term biosafety, biodistribution, immune responses, and potential neurotoxicity remain major barriers to clinical translation (13, 14).

In summary, while CAR-T therapy for GBM is still in its early clinical development and current outcomes remain modest, its biological rationale is strong and continued technological refinements hold promise for transforming the therapeutic landscape of this highly aggressive disease. However, many of the obstacles limiting the efficacy of conventional αβ CAR-T cells in GBM are intrinsic to their biology, prompting growing interest in alternative cellular platforms (15). Among these, γδ T-cell have emerged as a particularly attractive candidate due to their MHC-independent tumor recognition and innate-like response (16, 17), reduced risk of graft-versus-host disease, and ability to respond to stress-induced ligands abundantly expressed by GBM cells. The engineering of γδ CAR-T cells, therefore, represents a promising next-generation strategy to enhance the safety and efficacy of cellular immunotherapy in GBM (18).

## Conventional αβ CAR-T-cell therapy in GBM: preclinical and clinical evidence

Extensive preclinical and early clinical studies have investigated αβ CAR-T-cell therapy as a treatment strategy for GBM, primarily targeting tumor-associated antigens such as IL-13Rα2, EGFRvIII, HER2 and EphA2. In addition to these targets, GD2-directed αβ CAR-T cells have been investigated in highly translational preclinical GBM models, providing mechanistic insights into CAR-T cell dysfunction, antigen-driven exhaustion, and the impact of the CNS tumor microenvironment, rather than evidence of durable therapeutic efficacy (12). Preclinical work established the biological feasibility of CAR-T-mediated targeting of heterogeneous GBM cell populations, including stem-like compartments and tumor-initiating cells (19).

Translation into the clinical setting has confirmed feasibility and safety but revealed limited and often transient efficacy. Early-phase I trials of locoregionally delivered IL-13Rα2-directed CAR-T cells in patients with recurrent GBM demonstrated on-target antitumor activity, including radiographic tumor regression and disease stabilization, with a favorable safety profile and predominantly low-grade neurological adverse events (20, 21). Similarly, systemic administration of EGFRvIII-targeted CAR-T cells showed efficient tumor trafficking and antigen engagement but was frequently associated with rapid loss of target antigen expression and limited clinical responses, highlighting antigen escape as a dominant resistance mechanism (22).

More recent strategies have focused on addressing antigenic heterogeneity through multi-antigen targeting. In representative preclinical studies, multi-target CAR approaches (including trivalent constructs) improved tumor control across heterogeneous antigen-expression patterns and provided a rationale for mitigating immune escape (23). Consistently, a large phase I study of locoregional IL-13Rα2-directed CAR-T therapy in recurrent high-grade GBM patients confirmed sustained bioactivity and clinical responses in a subset of patients, including rare complete responses, although most responses remained limited in duration (24). Collectively, these studies demonstrate that αβ CAR-T cells can traffic to the central nervous system and mediate tumor-specific cytotoxicity in GBM, yet durable disease control remains uncommon, underscoring the need for alternative or complementary cellular platforms.

## Limits of αβ CAR-T-cell therapy

Despite the strong biological rationale and encouraging safety profile observed in early clinical studies, the therapeutic efficacy of conventional αβ CAR-T-cell therapy in GBM has remained modest. Multiple, interconnected barriers limit durable antitumor responses, many of which are intrinsic to both GBM biology and αβ T-cell-based cellular platforms.

One of the principal challenges is antigen heterogeneity and immune escape. GBM exhibits profound inter- and intra-tumoral heterogeneity, with variable expression of target antigens such as EGFRvIII, IL13Rα2, and HER2 across tumor regions and over time (15, 25). Clinical and preclinical studies have consistently shown that CAR-T-mediated pressure can rapidly select for antigen-negative tumor clones, resulting in immune escape and disease progression (20). This phenomenon is particularly problematic in GBM, where clonal evolution and phenotypic plasticity are driven by therapy-resistant GSCs, which may downregulate or entirely lack CAR target antigens.

A second major limitation relates to restricted trafficking and persistence of CAR-T cells within the CNS. Systemically administered CAR-T cells must overcome the BBB, an obstacle that limits efficient tumor infiltration (15, 25). Even when locoregional delivery strategies, such as intracavitary or intrathecal infusions, are used to bypass the BBB, CAR-T cells often exhibit limited persistence within the TME. Inadequate expansion and short-lived survival of infused cells have been repeatedly observed in clinical studies and are strongly associated with transient clinical responses (22).

The immunosuppressive tumor microenvironment of GBM further compromises CAR-T-cell function. GBM is characterized by high levels of inhibitory cytokines (e.g., TGF-β, IL-10), metabolic constraints, hypoxia and abundant immunosuppressive cell populations, including tumor-associated macrophages, microglia, regulatory T-cell and myeloid-derived suppressor cells (15, 25, 26). These factors collectively promote T-cell dysfunction, exhaustion, and apoptosis. Conventional αβ CAR-T cells, which heavily rely on sustained antigen stimulation and costimulatory signaling, are particularly vulnerable to exhaustion in this hostile milieu, leading to diminished cytotoxicity and cytokine production (20, 26).

In addition, antigen recognition by αβ CAR-T cells remains fundamentally limited to engineered target specificity, thereby constraining their ability to respond to antigen-loss variants or stress-induced changes in tumor cells (15, 25). Unlike innate-like lymphocytes, αβ CAR-T cells lack endogenous mechanisms to sense cellular stress signals that are abundantly expressed by GBM cells in response to hypoxia, DNA damage, and oncogenic transformation. This narrow recognition repertoire further contributes to therapeutic failure in the context of dynamic tumor evolution (25, 27).

Safety considerations also remain relevant, particularly in the CNS. While the incidence of severe cytokine release syndrome and immune effector cell–associated neurotoxicity syndrome has been relatively low in GBM CAR-T trials compared with hematologic malignancies, the risk of on-target/off-tumor neurotoxicity persists due to low-level expression of target antigens in normal brain tissue (22). Moreover, inflammatory responses within the confined intracranial space may result in cerebral edema and neurological deterioration, necessitating careful patient monitoring and dose optimization (22).

Advanced human-relevant experimental models have recently provided important mechanistic insights into CAR-T-cell dysfunction within the central nervous system tumor microenvironment. In a recent study employing H3.3K27M-altered diffuse midline GBM brainstem organoids, prolonged treatment with GD2-directed αβ CAR-T cells closely mirrored clinical observations, revealing heterogeneous antitumor responses, progressive functional exhaustion, and the emergence of distinct effector and dysfunctional CAR-T-cell states over time. Notably, the incorporation of brain-resident microglia into the organoid system demonstrated a central role for myeloid-mediated immunosuppression in limiting CAR-T cell efficacy, selectively impairing cytotoxic effector populations and promoting exhaustion-associated transcriptional programs. Collectively, these findings highlight how sustained antigen exposure and CNS-specific immunosuppressive cues critically shape CAR-T-cell fate and function, underscoring fundamental biological constraints of conventional αβ CAR-T platforms in brain tumors (12).

Collectively, these limitations underscore that the challenges facing CAR-T therapy in GBM are not solely technical but are deeply rooted in the biological constraints of αβ T-cell platforms and the unique tumor ecosystem of the CNS. While ongoing efforts aim to optimize antigen selection, CAR design, and delivery routes, these incremental refinements may be insufficient to overcome fundamental barriers such as antigen heterogeneity, immune escape, and T-cell dysfunction. These considerations have prompted increased interest in alternative cellular immunotherapy platforms that integrate innate immune recognition with adaptive cytotoxicity, thereby providing a broader and more resilient antitumor response within the GBM microenvironment.

The challenges associated with CAR-T cell therapy in brain tumors, including limited persistence, antigen heterogeneity, and the immunosuppressive tumor microenvironment, have been extensively reviewed elsewhere (26).

## γδ T-cell as a platform for CAR immunotherapy in GBM

**FIGURE 1:**
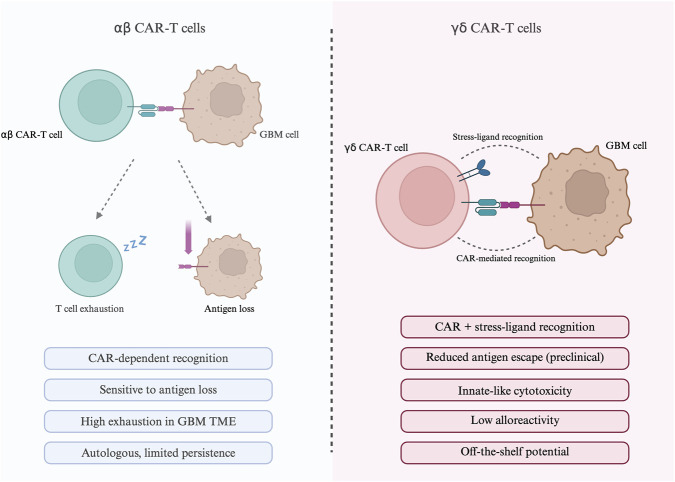
Comparison between αβ CAR-T cells and γδ CAR-T cells in GBM. αβ CAR-T cells rely solely on engineered antigen recognition and are constrained by antigen heterogeneity and exhaustion in the GBM microenvironment. γδ CAR-T cells integrate CAR-mediated targeting with innate stress-ligand recognition, allowing dual tumor sensing. Figure created using BioRender.

γδ T-cell constitute a distinct subset of T lymphocytes that bridge innate and adaptive immunity, displaying unique functional properties that differentiate them from conventional αβ T-cell (28). Unlike αβ T-cell, antigen recognition by γδ T-cell is MHC independent, enabling them to detect stress-induced ligands expressed by tumor cells without prior antigen presentation (29). This feature allows γδ T-cell to target heterogeneous tumor populations, irrespective on expression of specific tumor-associated antigens and may thus reduce the likelihood of immune escape observed with conventional CAR-T therapies. A schematic comparison between αβ and γδ CAR-T cells in the context of GBM is shown in Figure 1.

Human γδ T-cell are divided into Vδ1, Vγ9Vδ2 and Vδ3 subsets. Vδ1 T-cell are predominantly tissue-resident and enriched in epithelial and mucosal tissues, with emerging evidence supporting their presence in CNS tumors, whereas Vγ9Vδ2 T-cell circulate in peripheral blood and respond to phosphoantigens (30) generated by dysregulated metabolic pathways in tumor cells (31) or even induced by prior chemotherapy (32). Vδ3 T-cell constitute a minor and poorly characterized γδ T-cell subset, rarely detected in peripheral blood and mainly associated with specific tissue niches such as the liver.

The role of γδ T-cell in cancer immunity remains largely unexplored, and there is currently no evidence supporting their involvement in GBM or their use in CAR-based therapeutic strategies (31, 33).

In the context of GBM, γδ T-cell have been shown to engage multiple antitumor mechanisms, including stress-ligand recognition and cytokine-mediated cytotoxicity and to interact intricately with the immunosuppressive tumor microenvironment (34).

γδ T-cell exhibit innate-like cytotoxicity, mediated through multiple mechanisms: they express activating receptors such as NKG2D or NCR (like NKp46), secrete perforin and granzymes, and produce pro-inflammatory cytokines including IFN-γ and TNF-α, which collectively promote direct tumor lysis and recruitment of other immune effectors (35). Notably, γδ T-cell can recognize stress-induced ligands, such as MICA/B and ULBP family proteins, which are frequently upregulated in GBM cells and GSC, providing biologically relevant targets in a highly heterogeneous tumor (17, 35).

Beyond cytotoxicity, γδ T-cell exhibit extensive functional plasticity and multifunctionality, producing diverse cytokines and interacting with multiple immune subsets, features that underpin their potential in immunotherapeutic applications (18, 35, 36). The main preclinical and clinical studies investigating CAR-T cell therapy in GBM are summarized in Table 1. The interaction between γδ T-cell and the TME is complex; while they can exert antitumor functions, immunosuppressive signals can polarize them toward pro-tumoral phenotypes, emphasizing the need for strategies that reinforce antitumor polarization in GBM (34, 37, 38).

**TABLE 1:** Studies of CAR-T cell therapy in GBM.

Year	Study	Target	Population/Model	Clinical/Antitumor activity (compressed)	Toxicities	Outcome summary
2010	Ahmed et al., Clin Cancer Res (49)	HER2	Preclinical – GBM stem-cell xenograft models	Strong killing of HER2^+^ GBM stem-like cells; sustained tumor regression in mice	–	First proof-of-concept that HER2-directed CAR-T cells can target GBM stem cells
2012	Brown et al., Clin Cancer Res (19)	IL-13Rα2	Preclinical – GBM stem-like cells and orthotopic models	Efficient elimination of IL-13Rα2^+^ GBM stem-like cells and inhibition of tumor initiation	–	Validated IL-13Rα2 as a relevant GBM target
2013	Chow et al., Mol ther (50)	EphA2	Preclinical – *in vitro* + murine GBM models	Potent killing of EphA2^+^ GBM and GBM-initiating cells; tumor regression in mice	–	Supported EphA2 as an additional target for GBM CAR-T therapy
2014	Krebs et al., J Immunother Cancer (51)	IL-13Rα2 (mutein-based)	Preclinical – *in vitro*	IL-13 mutein CAR-T cells showed good activity against IL-13Rα2^+^ targets	–	Revealed cross-reactivity with IL-13Rα1, highlighting safety and specificity concerns
2015	Brown et al., Clin Cancer Res (21)	IL-13Rα2	Adult recurrent GBM - phase I, locoregional delivery	Local antitumor activity with transient radiographic regression and disease stabilization in several patients	Mostly grade 1–2 neurologic symptoms; no dose-limiting toxicity	Demonstrated feasibility and safety of intracavitary IL-13Rα2 CAR-T; responses were not durable
2016	Brown et al., N Engl J Med (20)	IL-13Rα2	Adult multifocal recurrent GBM – single-patient case	Near-complete regression of intracranial and spinal lesions; marked clinical improvement	No grade ≥3 CAR-related neurotoxicity reported	Deep response lasting ∼7.5 months before eventual progression
2017	O’Rourke et al., Sci Transl Med (22)	EGFRvIII	Adult recurrent GBM – phase I, intravenous infusion	On-target trafficking and reduction/loss of EGFRvIII expression; limited stable disease, rare objective responses	Mild infusion-related symptoms; no severe CRS or neurotoxicity	Showed feasibility of IV EGFRvIII CAR-T and identified antigen escape as a key resistance mechanism
2017	Ahmed et al., JAMA Oncol (49)	HER2 (virus-specific CAR-T)	Adult recurrent GBM/high-grade GBM - phase I, n ≈ 17	Several patients with prolonged stable disease; at least one partial response; median OS ∼11 months post infusion	Very safe profile; no evident on-target off-tumor HER2 toxicity; minimal CRS	Confirmed safety of systemic HER2 CAR-T in CNS tumors with modest clinical benefit
2018	Pituch et al., Mol Ther (52)	IL-13Rα2	Preclinical - syngeneic murine GBM models	Strong intratumoral activity, CAR-T persistence and remodeling of the tumor microenvironment	–	Supported the rationale for intratumoral/intraventricular IL-13Rα2 CAR-T delivery
2018	Bielamowicz et al., Neuro-Oncology (23)	Trivalent CAR (HER2/IL-13Rα2/EphA2)	Preclinical - heterogeneous GBM models	Eradicated tumor cells expressing different combinations of HER2, IL-13Rα2 and EphA2	–	Provided strong rationale for multi-antigen CAR-T approaches to overcome GBM heterogeneity
2022	Schmidts et al., Mol Ther Nucleic Acids (53)	EGFRvIII + IL-13Rα2 (tandem CAR)	Preclinical – heterogeneous GBM models	Robust activity against heterogeneous tumors; reduced emergence of antigen-negative escape variants	–	Supported bispecific CAR-T-cell strategies to counteract antigen escape
2024	Brown et al., Nat Med (24)	IL-13Rα2	Adult recurrent high-grade GBM (mainly GBM) - phase I, n = 65, locoregional delivery	Stable disease or better in ∼50% of evaluable patients; 2 partial responses and 1 complete response on protocol, plus 1 complete response off protocol	Treatment generally well tolerated; mostly grade 1–2 neurologic adverse events; no dose-limiting toxicity	Definitively confirmed safety and bioactivity of locoregional IL-13Rα2 CAR-T; clinical benefit present but often limited in duration
2025	Bessler et al., Nat Cancer (12)	GD2	Preclinical -human brainstem organoids modeling H3K27M-altered diffuse midline GBM	Prolonged GD2 CAR-T treatment induced heterogeneous antitumor responses, progressive functional exhaustion and emergence of dysfunctional CAR-T-cell states; microglia limited cytotoxic effector function	–	Provided human-relevant mechanistic evidence that CNS-specific immunosuppression and chronic antigen exposure constrain conventional αβ CAR-T-cell efficacy

The table summarizes key preclinical and clinical studies investigating CAR-T-cell therapies targeting GBM, including antigen specificity, experimental models, antitumor activity, toxicity profiles, and clinical outcomes. Together, these studies highlight both the therapeutic potential of CAR-T cells in GBM, and the major limitations associated with antigen heterogeneity, tumor immune escape, and treatment-related toxicity.

Importantly, γδ T-cell display a reduced risk of graft-versus-host disease (GVHD) in allogeneic settings, making them attractive candidates for off-the-shelf CAR engineering strategies (18). Their intrinsic cytotoxicity, combined with relative resistance to TME-mediated immunosuppression compared to αβ T-cell, suggests that γδ T-cell may overcome several barriers that limit the efficacy of standard CAR-T therapies in GBM.

Emerging evidence indicates that γδ T-cell can traffic to and infiltrate the CNS, including GBM lesions, and exert antitumor activity *in vivo* (31, 34). These features provide a strong rationale for developing γδ CAR-T-cell therapies, which combine the innate tumor-recognition properties of γδ T-cell with engineered antigen specificity, potentially enhancing cytotoxicity, persistence, and safety in the GBM microenvironment.

## γδ CAR-T cells in GBM immunotherapy: from engineering to preclinical and clinical applications

CAR-engineered γδ T-cell combine the intrinsic cytotoxicity and tissue tropism of γδ T-cell with the antigen-specific targeting conferred by chimeric antigen receptors (39–41). Moreover, γδ T-cell are considered a promising platform for CAR engineering due to their intrinsic antitumor activity and MHC-independent tumor recognition, potentially complementing CAR-mediated targeting (36–38). Among γδ T-cell subsets, Vδ1 T-cell are predominantly tissue-resident and have been detected in CNS tumors or inflammatory settings, whereas Vγ9Vδ2 cells circulate systemically, facilitating scalable *ex vivo* expansion for therapeutic applications (41). Subset selection is therefore a critical consideration for CAR engineering, as it impacts both trafficking to GBM lesions and functional durability within the immunosuppressive microenvironment (34, 41).

Emerging preclinical evidence suggests that CAR-engineered γδ T-cell may exert antitumor activity in GBM models, although GBM-specific data remain limited.

In a recent preclinical study, B7-H3-targeted CAR-modified Vγ9Vδ2 T-cell demonstrated significant cytotoxicity against GBM cell lines and patient-derived GSCs *in vitro*, accompanied by robust production of pro-inflammatory cytokines, including IFN-γ and TNF-α (42).

*In vivo*, γδ CAR-T cells were detectable within tumor-bearing xenograft models for a limited period following infusion, supporting their capacity for tumor infiltration and short-term persistence; however, direct comparative advantages over conventional αβ CAR-T cells in GBM remain to be established.

Clinically, the phase I INB-200 trial evaluated gene-modified autologous γδ T-cell (not CAR-engineered) in patients with newly diagnosed GBM, demonstrating feasibility, safety, and evidence of CNS trafficking, thereby providing proof-of-concept for γδ T-cell-based approaches rather than direct evidence for γδ CAR-T-cell efficacy (43). Broader CAR-T studies in high-grade GBMs provide contextual support for these findings, underscoring the translational potential of γδ CAR-T cells in solid CNS malignancies (44). Critical to this approach is the selection of target antigens: B7-H3, highly expressed in GBM cells and stem-like populations, has been validated as an effective CAR target, showing enhanced cytotoxicity in both preclinical and translational studies (45).

Overall, γδ CAR-T cells integrate innate-like immune recognition with CAR-mediated antigen specificity, representing a promising yet still largely experimental therapeutic platform for GBM. Preclinical and early clinical data demonstrate that they can achieve robust tumor killing, CNS infiltration, and persistence, providing a compelling rationale for further translational development within the constraints of current clinical evaluation.

Table 2 summarizes the available evidence supporting γδ CAR-T-cell strategies relevant to GBM. To date, this evidence remains limited and predominantly preclinical, with GBM-specific data confined to early experimental studies. Consequently, direct comparative data demonstrating advantages of γδ CAR-T cells over conventional αβ CAR-T cells in GBM are currently lacking. Nonetheless, the integration of innate-like tumor recognition with engineered antigen specificity positions γδ CAR-T cells as a promising, yet still early-stage, immunotherapeutic platform for GBM.

**TABLE 2:** Evidence supporting γδ CAR-T-cell strategies relevant to GBM.

Year	Study	CAR target	Model/Context	Key evidence	Interpretation for GBM
2018	Capsomidis et al., Molecular Therapy (46)	HER2	Solid tumor models (including CNS-relevant tumors)	CAR-engineered γδ T-cell retain innate cytotoxicity and show enhanced antitumor activity	First proof-of-concept γδ CAR-T study; GBM relevance indirect
2019	Fisher et al., Science Signaling (47)	Multiple (platform study)	Preclinical CAR engineering models	Reduced tonic CAR signaling and exhaustion compared to αβ CAR-T cells	Supports γδ CAR-T-cell platform advantages in hostile TMEs such as GBM
2017–2020	Seaman et al., Cancer Cell; Kabelitz et al., Cell Mol Immunol (33,48)	B7-H3 (target validation)	Solid tumors, including brain tumors (target/rationale studies)	B7-H3 broadly expressed in tumors; γδ T-cell exhibit innate antitumor properties	Provides biological rationale for B7-H3–targeted γδ CAR-T strategies, not direct evidence
2023	Wang et al., Journal of Translational Medicine (42)	B7-H3	GBM cell lines, patient-derived GSCs, xenograft models	CAR-modified Vγ9Vδ2 T-cell show enhanced cytotoxicity and cytokine production	GBM-specific preclinical evidence supporting γδ CAR-T strategies

To date, γδ CAR-T-cell therapies have been evaluated in only a few GBM-specific preclinical studies, while clinical data in this disease remain limited. The evidence summarized here derives from early GBM-focused experimental work, as well as from proof-of-concept γδ CAR-T-cell platform studies in solid tumors and target-validation literature supporting the applicability of γδ T-cell to central nervous system malignancies. Thus, γδ CAR-T cells approaches in GBM remain translationally promising but are still at an early stage of development.

## Conclusion

CAR-T-cell therapy constitutes a highly engineered immunological strategy that leverages the killing activity of T lymphocytes to selectively eradicate malignant cells. This approach is based on *ex vivo* genetic alteration of a patient’s own T-cell, enabling them to express synthetic antigen receptors specifically designed to detect and bind tumor-associated antigens (TAAs). While αβ CAR-T-cell immunotherapy holds great promise for treating GBM, several limitations and challenges remain to be addressed, including antigen loss, tumor heterogeneity, and immune suppression.

For these reasons, γδ CAR-T cells could represent a promising next-generation immunotherapy platform for GBM, integrating the innate, MHC-independent tumor-recognition properties of γδ T-cell with the antigen-specific targeting conferred by chimeric antigen receptors. Preclinical studies suggest cytotoxic activity against GBM models, including GSC-enriched systems; however, current evidence remains limited to early preclinical studies, and putative advantages in CNS trafficking, persistence, and resistance to immune escape have yet to be demonstrated in direct comparisons with conventional αβ CAR-T cells.

Nevertheless, the unique combination of innate-like immune surveillance, reduced alloreactivity, and suitability for off-the-shelf applications positions γδ CAR-T cells as an attractive and versatile cellular platform. Continued advances in γδ T-cell subset selection, CAR design, and antigen targeting may enable the development of more effective cellular immunotherapies for GBM.

## References

[1] Agosti E Garaba A Antonietti S Ius T Fontanella MM Zeppieri M CAR-T cells therapy in glioblastoma: a systematic review on molecular targets and treatment strategies. IJMS (2024) 25(13):7174. 10.3390/ijms25137174 39000281 PMC11241811

[2] Jang JK Pyo J Suh CH Park HS Chae YK Kim KW . Safety and efficacy of chimeric antigen receptor T-Cell therapy for glioblastoma: a systemic review and meta-analysis. Front Oncol (2022) 12:851877. 10.3389/fonc.2022.851877 35692797 PMC9178287

[3] Ma K Hu P . Chimeric antigen receptor T-Cell therapy for glioblastoma. Cancers. (2023) 15(23):5652. 10.3390/cancers15235652 38067356 PMC10705370

[4] Luksik AS Yazigi E Shah P Jackson CM . CAR T cell therapy in glioblastoma: overcoming challenges related to antigen expression. Cancers (Basel). (2023) 15(5):1414. 10.3390/cancers15051414 36900205 PMC10000604

[5] Franceschi E Tosoni A Bartolini S Mazzocchi V Fioravanti A Brandes AA . Treatment options for recurrent glioblastoma: pitfalls and future trends. Expert Rev Anticancer Ther (2009) 9(5):613–9. 10.1586/era.09.23 19445578

[6] Stupp R Taillibert S Kanner AA Kesari S Steinberg DM Toms SA Maintenance therapy with tumor-treating fields plus temozolomide vs temozolomide alone for glioblastoma: a randomized clinical trial. JAMA (2015) 314(23):2535–43. 10.1001/jama.2015.16669 26670971

[7] Wang H Xu T Jiang Y Xu H Yan Y Fu D The challenges and the promise of molecular targeted therapy in malignant gliomas. Neoplasia. (2015) 17(3):239–55. 10.1016/j.neo.2015.02.002 25810009 PMC4372648

[8] Weller M Van Den Bent M Tonn JC Stupp R Preusser M Cohen-Jonathan-Moyal E European association for neuro-oncology (EANO) guideline on the diagnosis and treatment of adult astrocytic and oligodendroglial gliomas. Lancet Oncol (2017) 18(6):6–e329. 10.1016/S1470-2045(17)30194-8 28483413

[9] Lacroix M Abi-Said D Fourney DR Gokaslan ZL Shi W DeMonte F A multivariate analysis of 416 patients with glioblastoma multiforme: prognosis, extent of resection, and survival. J Neurosurg (2001) 95(2):190–8. 10.3171/jns.2001.95.2.0190 11780887

[10] Delgado-López PD Corrales-García EM . Survival in glioblastoma: a review on the impact of treatment modalities. Clin Transl Oncol (2016) 18(11):1062–71. 10.1007/s12094-016-1497-x 26960561

[11] Omuro A DeAngelis LM . Glioblastoma and other malignant gliomas: a clinical review. JAMA (2013) 310(17):1842–50. 10.1001/jama.2013.280319 24193082

[12] Bessler N Wezenaar AKL Ariese HCR Honhoff C Dommann N Wehrens EJ *De novo* H3.3K27M-altered diffuse midline glioma in human brainstem organoids to dissect GD2 CAR T cell function. Nat Cancer (2026) 5:316–33. 10.1038/s43018-025-01084-0 PMC12948678 41492091

[13] Gao J Xia Z Gunasekar S Jiang C Karp JM Joshi N . Precision drug delivery to the central nervous system using engineered nanoparticles. Nat Rev Mater (2024) 9(8):567–88. 10.1038/s41578-024-00695-w

[14] Duan M Cao R Yang Y Chen X Liu L Ren B Blood–brain barrier conquest in glioblastoma nanomedicine: strategies, clinical advances, and emerging challenges. Cancers. (2024) 16(19):3300. 10.3390/cancers16193300 39409919 PMC11475686

[15] Lim M Xia Y Bettegowda C Weller M . Current state of immunotherapy for glioblastoma. Nat Rev Clin Oncol (2018) 15(7):422–42. 10.1038/s41571-018-0003-5 29643471

[16] Hayday AC . γδ T cells and the lymphoid stress-surveillance response. Immunity (2009) 31(2):184–96. 10.1016/j.immuni.2009.08.006 19699170

[17] Silva-Santos B Serre K Norell H . γδ T cells in cancer. Nat Rev Immunol (2015) 15(11):11–691. 10.1038/nri3904 26449179

[18] Fisher J Anderson J . Engineering approaches in human gamma Delta T cells for cancer immunotherapy. Front Immunol (2018) 9:1409. 10.3389/fimmu.2018.01409 29997614 PMC6028554

[19] Brown CE Starr R Aguilar B Shami AF Martinez C D’Apuzzo M Stem-like tumor-initiating cells isolated from IL13Rα2 expressing gliomas are targeted and killed by IL13-zetakine-redirected T cells. Clin Cancer Res (2012) 18(8):2199–209. 10.1158/1078-0432.CCR-11-1669 22407828 PMC3578382

[20] Brown CE Alizadeh D Starr R Weng L Wagner JR Naranjo A Regression of glioblastoma after chimeric antigen receptor T-Cell therapy. N Engl J Med (2016) 375(26):2561–9. 10.1056/NEJMoa1610497 28029927 PMC5390684

[21] Brown CE Badie B Barish ME Weng L Ostberg JR Chang WC Bioactivity and safety of IL13Rα2-Redirected chimeric antigen receptor CD8+ T cells in patients with recurrent glioblastoma. Clin Cancer Res (2015) 21(18):4062–72. 10.1158/1078-0432.CCR-15-0428 26059190 PMC4632968

[22] O’Rourke DM Nasrallah MP Desai A Melenhorst JJ Mansfield K Morrissette JJD A single dose of peripherally infused EGFRvIII-directed CAR T cells mediates antigen loss and induces adaptive resistance in patients with recurrent glioblastoma. Sci Transl Med (2017) 9(399):eaaa0984. 10.1126/scitranslmed.aaa0984 28724573 PMC5762203

[23] Bielamowicz K Fousek K Byrd TT Samaha H Mukherjee M Aware N Trivalent CAR T cells overcome interpatient antigenic variability in glioblastoma. Neuro Oncol (2018) 20(4):506–18. 10.1093/neuonc/nox182 29016929 PMC5909636

[24] Brown CE Hibbard JC Alizadeh D Blanchard MS Natri HM Wang D Locoregional delivery of IL-13Rα2-targeting CAR-T cells in recurrent high-grade glioma: a phase 1 trial. Nat Med (2024) 30(4):1001–12. 10.1038/s41591-024-02875-1 38454126 PMC11031404

[25] Maggs L Cattaneo G Dal AE Moghaddam AS Ferrone S . CAR T cell-based immunotherapy for the treatment of glioblastoma. Front Neurosci (2021) 15:662064. 10.3389/fnins.2021.662064 34113233 PMC8185049

[26] Woroniecka K Chongsathidkiet P Rhodin K Kemeny H Dechant C Farber SH T-Cell exhaustion signatures vary with tumor type and are severe in glioblastoma. Clin Cancer Res (2018) 24(17):4175–86. 10.1158/1078-0432.CCR-17-1846 29437767 PMC6081269

[27] Lim WA June CH . The principles of engineering immune cells to treat cancer. Cell. (2017) 168(4):724–40. 10.1016/j.cell.2017.01.016 28187291 PMC5553442

[28] Shekarkar AM Badami GD Di Caro M Tamburini B Fallo M Dieli C Deep immunoprofiling of large-scale tuberculosis dataset at single cell resolution reveals a CD81bright γδ T cell population associated with latency. Cells. (2024) 13(18):1529. 10.3390/cells13181529 39329713 PMC11430301

[29] Vantourout P Hayday A . Six-of-the-best: unique contributions of γδ T cells to immunology. Nat Rev Immunol (2013) 13(2):88–100. 10.1038/nri3384 23348415 PMC3951794

[30] Sireci G Espinosa E Di Sano C Dieli F Fournié JJ Salerno A . Differential activation of human gammadelta cells by nonpeptide phosphoantigens. Eur J Immunol (2001) 31(5):1628–35. 10.1002/1521-4141(200105)31:5<1628::AID-IMMU1628>3.0.CO 11465120

[31] Wu D Wu P Qiu F Wei Q Huang J . Human γδT-cell subsets and their involvement in tumor immunity. Cell Mol Immunol. (2017) 14(3):245–53. 10.1038/cmi.2016.55 27890919 PMC5360884

[32] Todaro M Meraviglia S Caccamo N Stassi G Dieli F . Combining conventional chemotherapy and γδ T cell-based immunotherapy to target cancer-initiating cells. OncoImmunology (2013) 2(9):e25821. 10.4161/onci.25821 24244907 PMC3825724

[33] Kabelitz D Serrano R Kouakanou L Peters C Kalyan S . Cancer immunotherapy with γδ T cells: many paths ahead of us. Cel Mol Immunol. (2020) 17(9):925–39. 10.1038/s41423-020-0504-x 32699351 PMC7609273

[34] Dieli C Maugeri R Corsale AM Di Simone M Avellone C Dieli F γδ T cells in glioblastoma multiforme: novel roles and therapeutic opportunities. Cancers. (2025) 17(16):2660. 10.3390/cancers17162660 40867289 PMC12384558

[35] Raulet DH Gasser S Gowen BG Deng W Jung H . Regulation of ligands for the NKG2D activating receptor. Annu Rev Immunol (2013) 31(1):413–41. 10.1146/annurev-immunol-032712-095951 23298206 PMC4244079

[36] Ligotti ME Accardi G Aiello A Calabrò A Caruso C Corsale AM Sicilian semi- and supercentenarians: age-related Tγδ cell immunophenotype contributes to longevity trait definition. Clin Exp Immunol (2024) 216(1):1–12. 10.1093/cei/uxad132 38066662 PMC10929699

[37] Lo Presti E Pizzolato G Corsale AM Caccamo N Sireci G Dieli F γδ T cells and tumor microenvironment: from immunosurveillance to tumor evasion. Front Immunol (2018) 9:1395. 10.3389/fimmu.2018.01395 29963061 PMC6013569

[38] Di Simone M Corsale AM Toia F Shekarkar Azgomi M Di Stefano AB Lo Presti E Tumor-infiltrating γδ T cells as targets of immune checkpoint blockade in melanoma. J Leukoc Biol (2024) 115(4):760–70. 10.1093/jleuko/qiae023 38324004

[39] Yazdanifar M Barbarito G Bertaina A Airoldi I . γδ T cells: the ideal tool for cancer immunotherapy. Cells. (2020) 9(5):5. 10.3390/cells9051305 32456316 PMC7290982

[40] Cieslak SG Shahbazi R . Gamma delta T cells and their immunotherapeutic potential in cancer. Biomark Res (2025) 13(1):51. 10.1186/s40364-025-00762-6 40148988 PMC11951843

[41] Zou C Zhao P Xiao Z Han X Fu F Fu L . γδ T cells in cancer immunotherapy. Oncotarget (2017) 8(5):8900–9. 10.18632/oncotarget.13051 27823972 PMC5352452

[42] Wang Y Ji N Zhang Y Chu J Pan C Zhang P B7H3-targeting chimeric antigen receptor modification enhances antitumor effect of Vγ9Vδ2 T cells in glioblastoma. J Transl Med (2023) 21(1):672. 10.1186/s12967-023-04514-8 37770968 PMC10537973

[43] Lobbous M Lamb LS Rochlin K Pillay T Ter Haak MA Nabors LB . INB-200: phase 1 study of gene-modified autologous gamma-delta (γδ) t cells in newly diagnosed glioblastoma multiforme (GBM) patients receiving maintenance temozolomide (TMZ). JCO Giugno (2025) 43(16_Suppl. l):2007. 10.1200/JCO.2025.43.16_suppl.2007

[44] Park S Maus MV Choi BD . CAR-T cell therapy for the treatment of adult high-grade gliomas. npj Precis Onc. (2024) 8(1):279. 10.1038/s41698-024-00753-0 PMC11659528 39702579

[45] Nehama D Di Ianni N Musio S Du H Patané M Pollo B B7-H3-redirected chimeric antigen receptor T cells target glioblastoma and neurospheres. EBioMedicine. (2019) 47:33–43. 10.1016/j.ebiom.2019.08.030 31466914 PMC6796553

[46] Capsomidis A Benthall G Van Acker HH Fisher J Kramer AM Abeln Z Chimeric antigen receptor-engineered human gamma Delta T cells: enhanced cytotoxicity with retention of cross presentation. Mol Ther (2018) 26(2):2–365. 10.1016/j.ymthe.2017.12.001 29310916 PMC5835118

[47] Fisher J Sharma R Don DW Barisa M Hurtado MO Abramowski P Engineering γδT cells limits tonic signaling associated with chimeric antigen receptors. Sci Signal (2019) 12(598):eaax1872. 10.1126/scisignal.aax1872 31506382 PMC7055420

[48] Seaman S Zhu Z Saha S Zhang XM Yang MY Hilton MB Eradication of tumors through simultaneous ablation of CD276/B7-H3-Positive tumor cells and tumor vasculature. Cancer Cell (2017) 31(4):501–15.e8. 10.1016/j.ccell.2017.03.005 28399408 PMC5458750

[49] Ahmed N Brawley V Hegde M Bielamowicz K Kalra M Landi D HER2-Specific chimeric antigen receptor-modified virus-specific T cells for progressive glioblastoma: a phase 1 dose-escalation trial. JAMA Oncol (2017) 3(8):1094–101. 10.1001/jamaoncol.2017.0184 28426845 PMC5747970

[50] Chow KKH Naik S Kakarla S Brawley VS Shaffer DR Yi Z T cells redirected to EphA2 for the immunotherapy of glioblastoma. Mol Ther (2013) 21(3):629–37. 10.1038/mt.2012.210 23070117 PMC3589173

[51] Krebs S Chow KKH Yi Z Rodriguez-Cruz T Hegde M Gerken C T cells redirected to interleukin-13Rα2 with interleukin-13 mutein--chimeric antigen receptors have anti-glioma activity but also recognize interleukin-13Rα1. Cytotherapy (2014) 16(8):1121–31. 10.1016/j.jcyt.2014.02.012 24841514 PMC4087074

[52] Pituch KC Miska J Krenciute G Panek WK Li G Rodriguez-Cruz T Adoptive transfer of IL13Rα2-Specific chimeric antigen receptor T cells creates a pro-inflammatory environment in glioblastoma. Mol Ther (2018) 26(4):986–95. 10.1016/j.ymthe.2018.02.001 29503195 PMC6079480

[53] Schmidts A Srivastava AA Ramapriyan R Bailey SR Bouffard AA Cahill DP Tandem chimeric antigen receptor (CAR) T cells targeting EGFRvIII and IL-13Rα2 are effective against heterogeneous glioblastoma. Neurooncol Adv (2023) 5(1):vdac185. 10.1093/noajnl/vdac185 36751672 PMC9896600

